# Association between Dietary Pattern and Periodontitis—A Cross-Sectional Study

**DOI:** 10.3390/nu13114167

**Published:** 2021-11-21

**Authors:** Ersin Altun, Carolin Walther, Katrin Borof, Elina Petersen, Berit Lieske, Dimitros Kasapoudis, Navid Jalilvand, Thomas Beikler, Bettina Jagemann, Birgit-Christiane Zyriax, Ghazal Aarabi

**Affiliations:** 1 Department of Periodontics, Preventive and Restorative Dentistry, University Medical Centre Hamburg-Eppendorf, 20246 Hamburg, Germany; ersin.altun@live.de (E.A.); k.borof@uke.de (K.B.); b.lieske@uke.de (B.L.); d.kasapoudis@outlook.com (D.K.); n.jalilvand@uke.de (N.J.); t.beikler@uke.de (T.B.); g.aarabi@uke.de (G.A.); 2 Department of Cardiology, University Heart and Vascular Center, 20246 Hamburg, Germany; e.petersen@uke.de; 3 Population Health Research Department, University Heart and Vascular Center, 20246 Hamburg, Germany; 4 Midwifery Science—Health Services Research and Prevention, Institute for Health Services Research in Dermatology and Nursing (IVDP), University Medical Center Hamburg-Eppendorf (UKE), 20246 Hamburg, Germany; b.jagemann@uke.de (B.J.); b.zyriax@uke.de (B.-C.Z.)

**Keywords:** dietary patterns, nutrition, oral health, periodontal disease, clinical attachment loss, DMFT

## Abstract

The aim of the study was to investigate the relationship between specific known dietary patterns and the prevalence of periodontal disease in a northern population-based cohort study. We evaluated data from 6209 participants of the Hamburg City Health Study (HCHS). The HCHS is a prospective cohort study and is registered at ClinicalTrial.gov (NCT03934957). Dietary intake was assessed with the food frequency questionnaire (FFQ2). Periodontal examination included probing depth, gingival recession, plaque index, and bleeding on probing. Descriptive analyses were stratified by periodontitis severity. Ordinal logistic regression models were used to determine the association. Ordinal regression analyses revealed a significant association between higher adherence to the DASH diet/Mediterranean diet and lower odds to be affected by periodontal diseases in an unadjusted model (OR: 0.92; 95% CI: 0.87, 0.97; *p* < 0.001/OR: 0.93; 95% CI: 0.91, 0.96; *p* < 0.001) and an adjusted model (age, sex, diabetes) (OR: 0.94; 95% CI: 0.89, 1.00; *p* < 0.0365/OR: 0.97; 95% CI: 0.94, 1.00; *p* < 0.0359). The current cross-sectional study identified a significant association between higher adherence to the DASH and Mediterranean diets and lower odds to be affected by periodontal diseases (irrespective of disease severity). Future randomized controlled trials are needed to evaluate to which extent macro- and micronutrition can affect periodontitis initiation/progression.

## 1. Introduction

According to the Fifth German Oral Health Survey, periodontitis affects up to 11.5 million people in Germany [1] and is one of the main reasons for tooth loss [2]. A primary risk factor for periodontitis is the accumulation of intraoral dysbiotic microflora at the tooth surface [3]. The host immune system responds to the bacterial colonization via destruction/degradation of the periodontal ligament and the surrounding bone structure [4]. Additionally, the host immune system is modulated by genetic, environmental, and behavioral factors [5].

It has been demonstrated that the reduction of oxidative stress in combination with an increased intake of antioxidants through diet has beneficial effects on gingival and periodontal inflammation due to the modulation of host immune responses [6,7]. A clinical controlled trial with 15 participants demonstrated that fewer carbohydrates, more omega-3 fatty acids, and more vitamins C and D, antioxidants, and fiber in the diet significantly reduce periodontal inflammatory parameters [8]. Higher levels of fiber and lower levels of fat in the diet improve markers of periodontitis in high-risk subjects [9].

The Dietary Approach to Stop Hypertension (DASH) and the Mediterranean diet consider these aspects. The Mediterranean diet refers to a traditional diet rich in plant-based foods, fish, and olive oil from the Mediterranean region. It is based on a low consumption of red meat and processed foods [10]. A high consumption of polyphenols through olive oil can increase the health benefits of the Mediterranean diet [11,12]. The DASH diet aims to control the blood pressure with a plant-based diet [13]. A Key aspect of this diet is a high intake of plant-based foods, low-fat dairy products, fish, and whole grains. Sodium, red meat, and processed foods should be reduced [14]. 

In 2013, those dietary approaches were initially recommended to prevent heart disease and stroke [15], as it was already well known that dietary habits influence the onset/progression of, e.g., coronary heart disease (CHD) and type 2 diabetes [16]. Unsaturated fatty acids, particularly polyunsaturated fatty acids (PUFA), and/or high-quality carbohydrates from whole grains, have been associated with a lower risk of CHD. In fact, high adherence to the Mediterranean diet or the DASH diet seems to have preventive effects against stroke and CHD [17,18]. 

Only a few studies investigated the effect of distinct dietary patterns on oral inflammation in large epidemiological settings [19,20,21,22] and, to the best of our knowledge, only one study specifically considered the DASH or the Mediterranean diet [23]. 

Consequently, the aim of the current cross-sectional study was to investigate the relationship between specific known dietary patterns (DASH and Mediterranean Diet) and the prevalence of periodontal disease in a northern population-based cohort study.

## 2. Materials and Methods

### 2.1. Subjects, Study Design, and Setting

In total, 6209 participants of the Hamburg City Health Study (HCHS) fitted the required inclusion criteria and were selected for analysis. The HCHS is a prospective cohort study conducted at the University Medical Center Hamburg-Eppendorf, with the aim to gain substantial knowledge on major chronic disease initiation and development [24]. Prior general and oral examination, all participants answered a questionnaire regarding their environmental conditions and lifestyle (e.g., physical condition and activity, dietary habits). The HCHS has been registered at ClinicalTrial.gov (NCT03934957). The Landesärztekammer Hamburg (State of Hamburg Chamber of Medical Practitioners, PV5131) approved the study protocol, and all participants signed an informed consent. The manuscript was written according to the STROBE Guidelines [25].

### 2.2. Sample Size Calculation

The periodontal cohort consisted of 1453 participants with none/mild periodontitis and 1176 participants with severe periodontitis. For the estimation of detectable effects due to healthy dietary patterns, we compared a change in the dietary score corresponding to the interquartile range [22], i.e., the half of the participants with “worse” dietary scores to the half with the “best” dietary scores. With a binomial response (mild/none vs. severe) and a significance level of 5%, we found that the study detected an effect of OR = 1.25 with 80% power. This effect is considered realistic and clinically relevant.

### 2.3. Assessment of Dietary Patterns

Initially developed for the European Prospective Investigation into Cancer and Nutrition (EPIC) study, the corresponding validated questionnaires were used to collect information on dietary intake [26]. The current version of the food frequency questionnaire (FFQ2) records the portion size and frequency of 102 food items eaten within the previous year [27]. Subsequently, energy intake, relevant food groups, and nutrients were assessed. 

### 2.4. DASH Adherence Score 

The scoring scheme adapted from Folsom et al. was used to determine adherence to the DASH diet [28]. Ten equally weighted food items (frequency consumption of fruits, dairy, grains, vegetables, nuts/seeds/legumes, meat/poultry/fish, and sweets obtained from raw data) and average daily nutrients intake (e.g., saturated fat, fat, sodium) were included. The DASH adherence score could be assessed for 9020 participants. For each dietary component, a score of 0–1 was given and summed across the 10 items [29] (Table 1; score of 10 = full adherence, score of 0 = nonadherence). A score from 0 to 3.5 was classified as low adherence, from 4 to 6.5 as medium adherence, and from 7 to 10 as high adherence. 

### 2.5. Mediterranean Adherence Score (MEDAS)

The validated German translation of the original MEDAS was used to determine adherence to the Mediterranean diet [30]. Twelve questions on food items (consumption of food groups and frequency by using raw data, mean daily intake of animal fat and vegetable oil) and additional 2 questions on characteristic food habits of the Mediterranean diet were included (Table 2). The MEDAS score could be assessed for 9020 participants and ranged from 0 to 14 points, with items scoring either 0 (condition not met) or 1 (adherent). A score from 0 to 4 was classified as low adherence, from 5 to 9 as medium adherence, and from 10 to 14 as high adherence.

### 2.6. Assessment of Dental Variables

The 6209 participants received a full dental examination (excluding wisdom teeth). Study nurses were trained and regularly calibrated. Calibration took place every two months and was performed by a trained dentist. Prior to oral examination, all participants were asked to undergo a necessary endocarditis prophylaxis. Oral examination included: probing depth (mm), gingival recession (mm), bleeding on probing (BOP) (6 sites per tooth; PCP-12 probe), and determination of the plaque index (4 sites per tooth). Subsequently, two secondary variables were calculated, i.e., DMFT (decayed, missing, filled teeth) and CAL (clinical attachment loss). All participants were then categorized in one out of three severity levels of periodontitis [31]: (1) no periodontitis/mild periodontitis, (2) moderate periodontitis, and (3) severe periodontitis.

### 2.7. Assessment of Additional Variables

For all participants, the following information was retrieved from the HCHS Database: gender, age (years), education (according to ISCED [32]), cardiovascular risk factors, i.e., BMI (in kg/m^2^), smoking (ever/non-smoking), diabetes (positive self-declaration and/or taking medication of the A10 group (insulin and analogues), and/or fasting glucose >126 mg/dL, not fasting glucose >200 mg/dL), and hypertension. High-sensitivity interleukin 6 (IL-6) and C-reactive protein (CRP) were assessed via established ELISA. Nutrition was reported as gram/day and included fiber, protein, fat, carbohydrates, and alcohol. Participants were asked about their physical activity level and hours of sport/week. 

### 2.8. Statistical Analysis

Descriptive analyses were stratified by periodontitis severity (and by gender in supplementary Tables S1–S3). Continuous variables are displayed as median and interquartile range [median (IQR)]. Consequently, categorical variables are displayed as absolute numbers and percentages [*n* (%)]. Differences within groups were tested using the Chi-squared test for categorical variables and the Kruskal–Wallis test for continuous variables. Dietary patterns were categorized into terciles, resulting in three groups: “Low”, “Medium”, and “High”. Ordinal logistic regression models were applied to determine the association between periodontitis (dependent variable) and dietary patterns (independent variable). Simplified logistic regression model is presented in supplementary material (supplementary Tables S4 and S5). Dietary patters were categorized using a numerical range. All statistical analyses were performed in R Studio Version 4.0.3 (Free Software Foundation’s GNU project) with standard 0.05 statistical significance.

## 3. Results

### 3.1. Descriptive Statistics Stratified According to Periodontitis Severity

Data from 6209 participants with fully completed periodontal examination were included. Participants with severe periodontitis were more likely men (60.9 vs. 39,6%), of higher age (66 vs. 59 years), with less frequent higher education (39.8 vs. 47.6 %) when compared to participants with none/mild periodontitis. Participants with severe periodontitis presented higher BMI values (26.4 vs. 25.5 kg/m^2^), were current smokers (25.1 vs. 16.2%), had more often diabetes (11.3 vs. 6.2 %), suffered from hypertension (72.5 vs. 54.8%), and differed in their CRP values (0.10 vs. 0.13 mg/l). Participants with severe periodontitis consumed (g/day) more proteins (72.64 vs. 69.20), more fat (91.41 vs. 86.81), more carbohydrates (198.21 vs. 195.19), and more alcohol (10.44 vs. 9.33) when compared to participants with none/mild periodontitis. Participants with severe periodontitis presented higher BOP Indices (21.05 vs. 2.08) and plaque indices (22.00 vs. 0.00) (Table 3).

Participants with low adherence to the DASH diet presented higher BOP Indices (8.70 vs. 7.14) and higher plaque indices (10.71 vs. 6.25) and were more affected by severe periodontitis (21.3 vs. 13.3%) when compared to participants with high adherence (Table 4).

We could detect a similar trend in participants with low adherence to the Mediterranean diet, with higher plaque index (10.42 vs. 3.57) and, overall, more cases with severe periodontitis (20.4 vs. 18.8%) when compared with participants with high adherence to the diet (Table 5).

### 3.2. Regression Analyses

Ordinal regression analyses revealed significant association between higher adherence to the DASH diet and lower odds to present periodontitis in an unadjusted model (OR: 0.92; 95% CI: 0.87, 0.97; *p* < 0.001), adjusted model 1 (age, sex, diabetes) (OR: 0.94; 95% CI: 0.89, 1.00; *p* < 0.0365), and adjusted model 2 (age, sex, and physical activity) (OR: 0.95; 95% CI: 0.89, 1.00; *p* < 0.0507) (Table 6).

We detected a similar trend for the Mediterranean diet with significant association between higher adherence to the diet and lower odds to present periodontitis in an unadjusted model (OR: 0.93; 95% CI: 0.91, 0.96; *p* < 0.001), adjusted model 1 (age, sex, diabetes) (OR: 0.97; 95% CI: 0.94, 1.00; *p* < 0.0359), and adjusted model 2 (age, sex, and physical activity) (OR: 0.97; 95% CI: 0.94, 1.00; *p* < 0.0515) (Table 7).

## 4. Discussion

In this cross-sectional study, dental and nutritional parameters were collected from 6209 individuals. Older men with higher cardiovascular burden appeared to be more affected by the severe form of periodontitis. Participants with poor adherence to the DASH diet and Mediterranean diet more often presented insufficient oral hygiene (higher BOP and plaque index) when compared to participants with high adherence to these diets. This trend was also perceptible in regression analyses: a higher adherence to the DASH diet as well as to the Mediterranean diet was significantly associated with lower odds to present periodontitis. 

A recently published cross-sectional study included rather young (mean age = 20.2 years) and healthy Moroccan individuals, with only 71 (6.6%) participants presenting periodontitis [23]. Logistic regression revealed no significant association between adherence to the Mediterranean diet and periodontitis, though the lack of a significant association might be because of (1) the small number of diseased participants and (2) the fact that the study population only consisted of undergraduate university students, who probably present overall better health and oral health literacy. 

Nielsen et al. analyzed data of 6052 adults participating in the National Health and Nutrition Examination Survey (NHANES 2011–2012) to investigate dietary fiber intake and periodontal disease prevalence [22]. The periodontal cohort was quite comparable to our cohort, with approximately 50% of female participants over 50 years of age and a majority of non-Hispanic white, who never smoked and graduated from college. Although the authors chose not to include the DASH diet or the Mediterranean diet specifically, they included fiber consumption, whole-grain consumption, and fruit and vegetable consumption. The authors reported a significant association between a low fiber consumption and moderate to severe periodontitis (referenced as mild–no periodontitis) compared with the association of periodontitis with a high fiber intake (OR: 1.30; 95% CI: 1.00, 1.69). 

The current case–control study design cannot address causality. Thus, the impact of the participants’ adherence to a diet can directly affect periodontitis via quite complex biological mechanisms (reviewed in detail [33]). In brief, carbohydrates can alter the microbial diversity and directly affect periodontal ligament cells. Saturated fatty acids are suspected to increase oxidative stress and thereby promote periodontal damage. 

However, participants’ adherence to a diet can also just act as confounding mediating factor. For example, behavioral patterns modify the association between dietary patterns and periodontitis. In fact, (1) women of all ages, unfortunately, are more often concerned about weight control and dieting, eating especially lower fat and lower sugar foods, as well as increasing the intake of fruits and vegetables [34], (2) men present lower health literacy, meaning lower understanding and application of health information for healthcare and disease prevention [35]. Consequently, it is possible that the significant association between lower adherence to dietary approaches and severe periodontitis is solely because of the accumulation of poor (oral) healthcare choices. 

Moreover, the DASH and Mediterranean diets were selected as examples of healthy plant-based diets recommended by international (AHA) and European guidelines (EAS). However, the two scores were not specifically developed for the HCHS study population. Therefore, the two dietary patterns may not fully reflect the specific dietary behaviors of the target population, although we observed a clear trend across all groups.”

## 5. Limitation

The population of the HCHS cohort is middle-aged white Caucasian, and our findings cannot be generalized to other ethnicities or to a much younger population. For periodontal classification, we used the reported gold standard: the AAP/CDC case definition for epidemiologic surveillance [31]. However, during the 2017 World Workshop on the Classification of Periodontal and Peri-Implant Diseases and Conditions, a new classification, “Staging and Grading”, was introduced [36]. Yet, this classification is not routinely used for epidemiological approaches. Thus, we set higher priority for the comparability of our study results. Lastly, the sample size of participants with severe periodontitis and high adherence to the DASH diet and the Mediterranean diet was small (<1%), which can be improved by, for example, purposeful over-sampling.

## 6. Conclusions

The current cross-sectional study identified a significant association between higher adherence to the DASH and Mediterranean diets and lower odds to be affected by periodontal diseases. Future randomized controlled trials are needed to evaluate to which extent macro- and micronutrition can affect periodontitis initiation/progression. 

## Figures and Tables

**Table 1:** Scoring criteria for DASH Dietary Adherence according to Epstein et al. [29].

Score Items	DASH-Component	Scoring
1	Total Grain	
	≥7 servings/day	1
	5–6 servings/day	0.5
	<5 servings/day	0
2	Vegetables	
	≥4 servings/day	1
	2–3 servings/day	0.5
	<2 servings/day	0
3	Fruits	
	≥4 servings/day	1
	2–3 servings/day	0.5
	<2 servings/day	0
4	Total dairy	
	≥2 servings/day	1
	1 servings/day	0.5
	<1 serving/day	0
5	Meat, poultry, and fish	
	≤2 servings/day	1
	3 servings/day	0.5
	≥4 serving/day	0
6	Nuts, seeds, and legumes	
	≥4 servings/day	1
	2–3 servings/day	0.5
	<2 servings/day	0
7	% kcal from fat	
	≤27%	1
	≥28 ≤ 29%	0.5
	≥30%	0
8	% kcal from saturated fat	
	≤6%	1
	≤7 ≥8%	0.5
	≥9%	0
9	Sweets	
	≤5 servings/week	1
	6–7 servings/week	0.5
	≥8 serving/week	0
10	Sodium	
	≤2.400 mg/day	1
	2.400–3.000 mg/day	0.5
	>3.000 mg/day	0

**Table 2:** Scoring criteria for Mediterranean Dietary score according to Hebestreit et al. [30].

Score Items	MEDAS Question	Data Recorded by FFQ 1 Point Given, If …
1	Do you use olive oil as the principal source of fat for cooking?	use of olive oil for the preparation of at least 2 of the following groceries: salad, vegetable, meat/fish
2	How much olive oil do you consume per day (including that used in frying, salads, meals eaten away from home, etc.)?	based on FFQ calculation, if >48 g vegetable oil per day
3	How many servings of vegetables do you consume per day?	based on FFQ calculation, if ≥2 portions of vegetables per day (including raw and cooked vegetables, salad, olives, mushrooms, except potatoes and legumes)
4	How many pieces of fruit (including fresh-squeezed juice) do you consume per day?	based on FFQ calculation, if ≥3 portions of fruit (including fruit, mixed fruit, fruit salad, mixed stewed fruit, and fruit juices, excluding sweetened beverages)
5	How many servings of red meat, hamburger, or sausages do you consume per day?	based on FFQ calculation, if <100 g red meat (e.g., beef, veal, pork, lamb) and processed meat products
6	How many servings (12 g) of butter, margarine, or cream do you consume per day?	based on FFQ calculation, if <1 portion butter, margarine, and cream and other animal fat
7	How many carbonated and/or sugar-sweetened beverages do you consume per day?	based on FFQ calculation, sugar-sweetened beverages <1 portion per day (including lemonade and colas)
8	Do you drink wine? How much do you consume per week?	based on FFQ calculation, if ≥7 portions wine (red and white wine)
9	How many servings of pulses do you consume per week?	≥3 portions pulses (e.g., beans, lentils, peas, chickpeas)
10	How many servings of fish/seafood do you consume per week?	based on FFQ calculation, if ≥3 portions fish, fish products, and seafood per week
11	How many times do you consume commercial (not homemade) pastry such as cookies or cake per week?	based on FFQ calculation, if <3 portions cakes, chocolate, cookies, sweets with and without chocolate per week
12	How many times do you consume nuts per week?	based on FFQ calculation, if ≥3 portions nuts per week
13	Do you prefer to eat chicken, turkey, or rabbit instead of beef, pork, hamburgers, or sausages?	Based on FFQ calculation, if g white meat (e.g., chicken, hen, and other poultry) > g red meat (e.g., beef, veal, pork, lamb, and processed meat products)
14	How many times per week do you consume boiled vegetables, pasta, rice, or other dishes with a sauce of tomato, garlic, onion, or leeks sautéed in olive oil?	> 1–2 times/week tomato sauce

**Table 3:** Baseline characteristics.

	None/Mild Periodontitis	Moderate Periodontitis	Severe Periodontitis	*p*-Value for Trend
*n* (%), median; IQR, * mean/SD	1453	3580	1176	
DEMOGRAPHICS				
Female sex	878 (60.4)	1814 (50.7)	460 (39.1)	<0.001
Age	59.00; 52.00, 66.00	63.00; 55.00, 69.00	66.00; 59.00, 71.00	<0.001
Education				0.001
Low	37 (2.7)	112 (3.4)	44 (4.1)	
Medium	678 (49.7)	1701 (51.0)	609 (56.2)	
High	650 (47.6)	1521 (45.6)	431 (39.8)	
CARDIOVASCULAR RISK				
BMI	25.56; 23.01, 28.67	26.02; 23.55, 29.01	26.44; 24.11, 29.65	<0.001
Smoking	235 (16.2)	608 (17.1)	293 (25.1)	<0.001
Diabetes	85 (6.2)	242 (7.4)	122 (11.3)	<0.001
Hypertension	768 (54.8)	2266 (66.3)	810 (72.5)	<0.001
LABORATORIES				
IL6	1.45; 1.01, 2.04	1.55; 1.15, 2.16	1.77; 1.33, 2.63	<0.001
CRP	0.10; 0.06, 0.23	0.11; 0.06, 0.25	0.13; 0.07, 0.30	<0.001
NUTRITION				
Total energy (kcal/day)	1989; 1569, 2543	2048; 1629, 2594	2069; 1639, 2602	0.057
Fibre (g/day)	18.88; 14.91, 24.29	18.94; 14.98, 24.07	18.23; 14.46, 23.41	0.023
Protein (g/day)	69.20; 54.97, 87.42	71.35; 56.41, 90.35	72.64; 57.27, 91.34	0.003
Fat (g/day)	86.81; 68.39, 113.25	90.04; 70.98, 114.56	91.41; 70.64, 115.76	0.023
Carbohydrates (g/day)	195.19; 153.19, 254.80	198.36; 153.70, 255.73	198.21; 150.12, 248.81	0.554
Alcohol (g/day)	9.33; 2.69, 21.52	9.38; 2.70, 22.94	10.44; 2.78, 26.41	0.140
Saccharides	89.21; 68.76, 116.66	89.81; 68.06, 119.89	87.83; 66.93, 114.71	0.187
DASH diet *	4.55/1.06	4.51/1.08	4.39/1.07	0.001
Mediterranean Diet *	4.77/1.92	4.58/1.88	4.38/1.87	<0.001
PHYSICAL ACTIVITY				
Physical activity	983 (75.5)	2286 (72.4)	657 (64.7)	<0.001
Sport (h/week)	2.00; [0.38, 4.00]	2.00; 0.00, 4.00	2.00; 0.00, 3.50	<0.001
DENTAL VARIABLES				
DMFT index	17.00; 14.00, 21.00	19.00; 16.00, 23.00	21.00; 17.00, 24.25	<0.001
BOP index	2.08; 0.00, 7.14	8.33; 2.17, 19.23	21.05; 9.26, 41.67	<0.001
Plaque index	0.00; 0.00, 10.71	8.93; 0.00, 27.78	22.00; 5.77, 54.76	<0.001

Abbreviations: BMI = Body Mass Index, BOP Index = Bleeding on Probing Index, DMFT Index = Decayed, Missing, Filled, Teeth Index, CRP = High-sensitivity C-reactive protein, IL6 = Interleukin 6, PA = periodontitis, * values for adherence score are displayed as Mean and Standard deviation.

**Table 4:** Baseline dental characteristics stratified according to adherence to the DASH diet.

	DASH Score			
	Low	Medium	High	*p*-Value
*n* *	2259	6718	43	
DMFT index	20.00; 16.00, 24.00	19.00; 16.00, 23.00	19.00; 14.00, 22.00	<0.001
BOP index	8.70; 2.00, 22.00	7.50; 1.92, 19.44	7.14; 2.07, 14.98	0.081
Plaque index	10.71; 0.00, 35.36	7.41; 0.00, 26.92	6.25; 0.00, 29.63	<0.001
Periodontitis				0.046
None/mild	304 (22.2)	1014 (23.9)	9 (30.0)	
Moderate	775 (56.5)	2472 (58.3)	17 (56.7)	
Severe	292 (21.3)	755 (17.8)	4 (13.3)	

Abbreviations: BOP Index = Bleeding on Probing Index, DMFT Index = Decayed, Missing, Filled, Teeth Index, *n* * = Adherence score could be assessed for 9020 participants–not all columns sum up to 100%, because of missing values for different variables.

**Table 5:** Baseline dental characteristics stratified according to adherence to the Mediterranean diet.

	Mediterranean Score			
	Low	Medium	High	*p*-Value
*n* *	4589	4380	51	
DMFT index	20.00; 16.00, 23.00	19.00; 15.00, 23.00	21.00; 15.00, 25.00	<0.001
BOP index	8.93; 2.00, 22.36	7.14; 1.85, 18.18	9.99; 1.34, 25.45	<0.001
Plaque index	10.42; 0.00, 33.33	6.82; 0.00, 25.00	3.57; 0.00, 23.30	<0.001
Periodontitis				0.010
None/mild	644 (22.5)	676 (24.6)	7 (21.9)	
Moderate	1633 (57.1)	1612 (58.6)	19 (59.4)	
Severe	584 (20.4)	461 (16.8)	6 (18.8)	

Abbreviations: BOP Index = Bleeding on Probing Index, DMFT Index = Decayed, Missing, Filled, Teeth Index, *n* * = Adherence score could be assessed for 9020 participants–not al columns sum up to 100%, because of missing values for different variables.

**Table 6:** Ordinal logistic regression: outcome periodontitis, exposure DASH diet.

Variable	Units	Odds Ratio	95% CI	*p*-Value
DASH diet		0.92	0.87; 0.97	<0.001
DASH diet		0.94	0.88; 0.99	0.0122
Age		1.05	1.05; 1.06	<0.001
Sex	Male	Ref		
	Female	0.64	0.53; 0.75	<0.001
DASH diet		0.94	0.89; 1.00	0.0365
Age		1.05	1.04; 1.06	<0.001
Sex	Male	Ref		
	Female	0.65	0.53; 0.76	<0.001
Diabetes	No	Ref		
	Yes	1.13	0.92; 1.34	0.2494
DASH diet		0.95	0.89; 1.00	0.0507
Age		1.05	1.04; 1.06	<0.001
Sex	Male	Ref		
	Female	0.64	0.53; 0.76	<0.001
Physical activity	No	Ref		
	Yes	0.81	0.69; 0.94	<0.001

Abbreviations: DASH = Dietary Approach to Stop Hypertension, CI = confidence interval. Unadjusted and stepwise adjusted for age, sex, diabetes, and physical activity.

**Table 7:** Ordinal logistic regression: outcome periodontitis, exposure Mediterranean Diet.

**Variable**	**Units**	**Odds Ratio**	**95% CI**	***p*** **-Value**
Mediterranean Diet		0.93	0.91; 0.96	<0.001
Mediterranean Diet		0.96	0.94; 0.99	0.0157
Age		1.05	1.04; 1.06	<0.001
Sex	Male	Ref		
	Female	0.64	0.53; 0.75	<0.001
Mediterranean Diet		0.97	0.94; 1.00	0.0359
Age		1.05	1.04; 1.06	<0.001
Sex	Male	Ref		
	Female	0.65	0.53; 0.76	<0.001
Diabetes	No	Ref		
	Yes	1.13	0.93; 1.34	0.2368
Mediterranean Diet		0.97	0.94; 1.00	0.0515
Age		1.05	1.04; 1.06	<0.001
Sex	Male	Ref		
	Female	0.64	0.53; 0.76	<0.001
Physical activity	No	Ref		
	Yes	0.81	0.69; 0.93	<0.001

Abbreviations: CI = confidence interval. Unadjusted and stepwise adjusted for age, sex, diabetes, and physical activity.

## Supplementary Materials

The following are available online at https://www.mdpi.com/article/10.3390/nu13114167/s1, Table S1: Baseline characteristics, Table S2: Baseline dental characteristics stratified according to DASH Diet, Table S3: Baseline dental characteristics stratified according to Mediterranean Diet, Table S4: Logistic regression: outcome binary variable periodontitis (no vs. severe periodontitis), exposure DASH Diet, Table S5: Logistic regression: outcome binary variable periodontitis (no vs. severe periodontitis), exposure Mediterranean Diet.
