# Supplementary material for: Association between Dietary Pattern and Periodontitis—A Cross-Sectional Study
Source: Nutrients. 2021 Nov 21;13(11):4167. doi: 10.3390/nu13114167 (PMC8621734; doi:10.3390/nu13114167)
Supplement: Supplementary file 1 [file nutrients-13-04167-s001.zip › nutrients-1459121-supplementary.pdf]

**Table S1.** Baseline characteristics.

| | Male | Female | p-value for Trend |
|---|---|---|---|
| n (%), median [IQR], * mean/SD | 4892 | 5108 | |
| DEMOGRAPHICS | | | |
| Age | 64.00 [56.00,70.00] | 62.00 [55.00,69.00] | <0.001 |
| Education | | | <0.001 |
| Low | 149 (3.2) | 295 (6.1) | |
| Medium | 1990 (43.2) | 2840 (58.8) | |
| High | 2465 (53.5) | 1698 (35.1) | |
| CARDIOVASCULAR RISK | | | |
| BMI | 26.68 [24.50,29.44] | 25.30 [22.63,28.89] | <0.001 |
| Smoking | 981 (20.2) | 997 (19.6) | <0.505 |
| Diabetes | 490 (10.8) | 304 (6.5) | <0.001 |
| Hypertension | 3398 (73.1) | 2903 (59.5) | <0.001 |
| LABORATORIES | | | |
| IL6 | 1.67 [1.22,2.38] | 1.55 [1.12,2.25] | <0.001 |
| CRP | 0.12 [0.06,0.26] | 0.12 [0.06,0.27] | 0.334 |
| NUTRITION | | | |
| Total energy (kcal/day) | 2391.24 [1937.93,2935.64] | 1761.34 [1443.94,2133.69] | <0.001 |
| Fibre (g/day) | 20.42 [15.74,25.78] | 17.32 [13.91,21.65] | <0.001 |
| Protein (g/day) | 84.20 [67.54,102.98] | 61.76 [50.00,74.99] | <0.001 |
| Fat (g/day) | 104.78 [84.16,131.06] | 77.38 [62.57,94.87] | <0.001 |
| Carbohydrates (g/day) | 227.70 [177.25,287.87] | 172.00 [137.68,216.20] | <0.001 |
| Alcohol (g/day) | 13.70 [4.09,29.45] | 6.21 [1.62,16.73] | <0.001 |
| Saccharides | 96.60 [72.05,128.16] | 83.95 [64.05,109.81] | <0.001 |
| DASH diet | 4.00 [3.50,5.00] | 5.00 [4.00,5.50] | <0.001 |
| Mediterranean Diet | 4.00 [3.00,5.00] | 5.00 [4.00,6.00] | <0.001 |
| PHYSICAL ACTIVITY | | | |
| Physical activity | 2826 (66.0) | 3239 (73.1) | <0.001 |
| Sport (h/week) | 2.00 [0.00,4.00] | 2.00 [0.00,4.00] | 0.001 |
| DENTAL VARIABLES | | | |
| DMFT index | 19.00 [15.00,24.00] | 20.00 [16.00,23.00] | 0.741 |
| BOP index | 8.70 [2.00,21.43] | 7.41 [1.85,19.23] | 0.001 |
| Plaque index | 12.50 [0.00,38.02] | 5.56 [0.00,23.08] | <0.001 |
| PERIODONTITIS | | | <0.001 |
| None/mild PA | 575 (18.8) | 878 (27.9) | |
| Moderate PA | 1766 (57.8) | 1814 (57.6) | |
| Severe PA | 716 (23.4) | 460 (14.6) | |

**Abbreviations:** BMI = Body Mass Index, BOP Index = Bleeding on Probing Index, DMFT Index = Decayed, Missing, Filled, Teeth Index, CRP = High sensitive c-reactive protein, IL6 = Interleukin 6, PA = periodontitis.

**Table S2.** Baseline dental characteristics stratified according to DASH Diet.

| | Low:Male | High:Male | Medium:Male | Low:Female | High:Female | Medium:Female |
|---|---|---|---|---|---|---|
| **n*** | 1651 | 16 | 2736 | 608 | 27 | 3982 |
| **DMFT-index** | 20.00 [16.00,24.00] | 18.50 [14.00, 22.00] | 19.00 [15.00,23.00] | 20.00 [16.00,24.00] | 19.00 [13.75,21.25] | 19.00 [16.00,23.00] |
| **BOP index** | 8.93 [2.08,23.08] | 4.95 [3.70, 12.01] | 8.33 [2.00,20.28] | 7.14 [1.82,20.83] | 8.33 [1.44,15.43] | 7.14 [1.85,18.75] |
| **Plaque index** | 12.50 [0.00,39.29] | 6.33 [0.00, 40.74] | 11.54 [0.00,35.19] | 7.14 [0.00,23.80] | 6.25 [0.00,23.08] | 5.26 [0.00,22.73] |
| **Periodontitis (%)** | | | | | | |
| **None/mild** | 199 (19.8) | 2 (20.0) | 333 (18.9) | 105 (28.8) | 7 (35.0) | 681 (27.5) |
| **Moderate** | 572 (56.9) | 6 (60.0) | 1026 (58.2) | 203 (55.6) | 11 (55.0) | 1446 (58.4) |
| **Severe** | 235 (23.4) | 2 (20.0) | 405 (23.0) | 57 (15.6) | 2 (10.0) | 350 (14.1) |

**Abbreviations:** BOP Index = Bleeding on Probing Index, DMFT Index = Decayed, Missing, Filled, Teeth Index, n* = Adherence score could be assessed for 9020 participants – every column does not sum up to 100%, because of missing values for different variables.

**Table S3.** Baseline dental characteristics stratified according to Mediterranean Diet.

| | Low:Male | High:Male | Medium:Male | Low:Female | High:Female | Medium:Female |
|---|---|---|---|---|---|---|
| **n*** | 2904 | 10 | 1489 | 1685 | 41 | 2891 |
| **DMFT-index** | 20.00 [16.00,24.00] | 15.00 [13.00,19.00] | 19.00 [15.00,23.00] | 20.00 [16.00,23.00] | 22.50 [16.75,26.25] | 19.00 [16.00,23.00] |
| **BOP index** | 9.09 [2.08,23.21] | 9.09 [4.91,13.85] | 7.41 [1.92,18.42] | 7.69 [1.92,21.15] | 11.61 [0.45,26.34] | 7.14 [1.85,18.00] |
| **Plaque index** | 12.50 [0.00,38.46] | 3.57 [0.00,54.63] | 10.71 [0.00,32.69] | 6.25 [0.00,25.00] | 3.57 [0.00,22.73] | 4.76 [0.00,21.15] |
| **Periodontitis (%)** | | | | | | |
| **None/mild** | 359 (19.7) | 2 (33.3) | 173 (18.2) | 285 (27.5) | 5 (19.2) | 503 (28.0) |
| **Moderate** | 1040 (57.0) | 2 (33.3) | 562 (59.2) | 593 (57.2) | 17 (65.4) | 1050 (58.4) |
| **Severe** | 425 (23.3) | 2 (33.3) | 215 (22.6) | 159 (15.3) | 4 (15.4) | 246 (13.7) |

**Abbreviations:** BOP Index = Bleeding on Probing Index, DMFT Index = Decayed, Missing, Filled, Teeth Index, n* = Adherence score could be assessed for 9020 participants – every column does not sum up to 100%, because of missing values for different variables.

**Table S4.** Logistic regression: outcome binary variable periodontitis (no vs. severe periodontitis), exposure DASH Diet.

| Variable | Units | Odds Ratio | 95% CI | p-value |
|---|---|---|---|---|
| **DASH diet** | | 0.90 | [0.82;0.98] | 0.0201 |
| **Age** | | 1.08 | [1.07;1.10] | <0.001 |
| **Sex** | Male | Ref | | |
| | Female | 0.48 | [0.40;0.58] | <0.001 |
| **DASH diet** | | 0.91 | [0.83;0.99] | 0.0341 |
| **Age** | | | | |
| | 45-54 | Ref | | |
| | 55-64 | 2.55 | [2.01;3.24] | <0.001 |
| | 65-74 | 4.71 | [3.70;5.98] | <0.001 |
| | 75+ | 8.55 | [5.55;13.20] | <0.001 |
| **Sex** | Male | Ref | | |
| | Female | 0.49 | [0.40;0.58] | <0.001 |

**Abbbreviations**: DASH = Dietary Approach to Stop Hypertension, CI = confidence

**Table S5.** Logistic regression: outcome binary variable periodontitis (no vs. severe periodontitis), exposure Mediterranean Diet.

| Variable | Units | Odds Ratio | 95% CI | p-value |
|---|---|---|---|---|
| **Mediterranean Diet** | | 0.94 | [0.89;0.98] | 0.00845 |
| **Age** | | 1.08 | [1.07;1.10] | <0.001 |
| **Sex** | Male | Ref | | |
| | Female | 0.49 | [0.41;0.59] | <0.001 |
| **Mediterranean Diet** | | 0.94 | [0.89;0.98] | 0.0093 |
| **Age** | | | | |
| | 45-54 | Ref | | |
| | 55-64 | 2.53 | [1.99;3.22] | <0.001 |
| | 65-74 | 4.66 | [3.67;5.92] | <0.001 |
| | 75+ | 8.45 | [5.49;13.02] | <0.001 |
| **Sex** | Male | Ref | | |
| | Female | 0.49 | [0.41;0.60] | <0.001 |

**Abbbreviations**: CI = confidence
